# Perfusion vector—a new method to quantify myocardial perfusion scintigraphy images: a simulation study with validation in patients

**DOI:** 10.1186/s13550-015-0121-3

**Published:** 2015-08-14

**Authors:** David Minarik, Martin Senneby, Per Wollmer, Alva Mansten, Karl Sjöstrand, Lars Edenbrandt, Elin Trägårdh

**Affiliations:** Radiation Physics, Skåne University Hospital, Lund University, Malmö, Sweden; Department of Translational Medicine, Clinical Physiology and Nuclear Medicine Unit, Lund University, Inga Marie Nilssons gata 49, 205 05 Malmö, Sweden; Informatics and Mathematical Modeling, Technical University of Denmark, Copenhagen, Denmark; EXINI Diagnostics AB, Lund, Sweden

**Keywords:** SPECT, Ischemic heart disease, Quantification, Automated method

## Abstract

**Background:**

The interpretation of myocardial perfusion scintigraphy (MPS) largely relies on visual assessment by the physician of the localization and extent of a perfusion defect. The aim of this study was to introduce the concept of the perfusion vector as a new objective quantitative method for further assisting the visual interpretation and to test the concept using simulated MPS images as well as patients.

**Methods:**

The perfusion vector is based on calculating the difference between the anatomical centroid and the perfusion center of gravity of the left ventricle. Simulated MPS images were obtained using the SIMIND Monte Carlo program together with XCAT phantom. Four different-sized anterior and four lateral defects were simulated, and perfusion vector components *x*-, *y*-, and *z*-axes were calculated. For the patient study, 40 normal and 80 abnormal studies were included. Perfusion vectors were compared between normal and abnormal (apical, inferior, anterior, and lateral ischemia or infarction) studies and also correlated to the defect size.

**Results:**

For simulated anterior defects, the stress perfusion vector component on the *y*-axis (anterior-inferior direction) increased in proportion to the defect size. For the simulated lateral defects, the stress perfusion vector component on the *x*-axis (septal-lateral direction) decreased in proportion to the defect size. When comparing normal and abnormal patients, there was a statistically significant difference for the stress perfusion vector on the *x*-axis for apical and lateral defects; on the *y*-axis for apical, inferior, and lateral defects; and on the *z*-axis (basal-apical direction) for apical, anterior, and lateral defects. A significant difference was shown for the difference vector magnitude (stress/rest) between normal and ischemic patients (*p* = 0.001) but not for patients with infarction. The correlation between the defect size and stress vector magnitude was also found to be significant (*p* < 0.001).

**Conclusions:**

The concept of the perfusion vector introduced in this study is shown to have potential in assisting the visual interpretation in MPS studies. Further studies are needed to validate the concept in patients.

## Background

Myocardial perfusion scintigraphy (MPS) is a widely used diagnostic method for the management of patients with suspected or known ischemic heart disease [1–5]. Single-photon emission computed tomography (SPECT) is used to generate three-dimensional images of radioactive tracer distribution in the left ventricular myocardium.

Software packages for quantification of perfusion data and computer-aided diagnosis systems have been developed in order to make the interpretation of MPS studies more standardized, both regarding left ventricular function and regarding perfusion data. For perfusion data, a 17- or 20-segment model is often used that evaluates both the extent and severity of perfusion defects during stress and rest, generating a summed stress score (SSS) and a summed rest score (SRS). The difference (summed difference score (SDS)) indicates the degree of ischemia. However, it has been shown that there is a considerable variability in the scoring values between different software packages [6–9], and this variability will affect the calculated amount of ischemia. Therefore, the physician interpreting the study will use both visual assessment and scoring values for the determination of the localization and amount of ischemia. Visual interpretation has on the other hand been shown to be subject to rather large inter- and intra-observer variability [10]. Hence, there is a need for new objective quantitative methods to be used in adjunct to the visual analysis to facilitate a correct diagnosis.

In this study, we have created a method that could assist the visual interpretation in MPS studies. The concept of the perfusion vector introduced here is based on the centroid. The centroid is the geometric center of gravity in a three-dimensional figure, in this case, the left ventricle. The left ventricle is outlined from SPECT images, and the anatomical and perfusion centroids are calculated. The location of the perfusion center of gravity will depend on the relative perfusion in the left ventricle. If the perfusion is normal throughout the left ventricle, the perfusion center of gravity and the anatomical centroid will be located close to each other. The difference between the anatomical and perfusion centroids is called the perfusion vector and will be small if the perfusion is normal. If the perfusion is not homogenous, the perfusion center of gravity will deviate from the anatomical centroid and this will result in a larger perfusion vector. The aim of this study was to test the concept of the perfusion vector in simulated MPS images and then to further validate the method in a clinical setting.

## Methods

### Perfusion vector concept

**Fig. 1:**
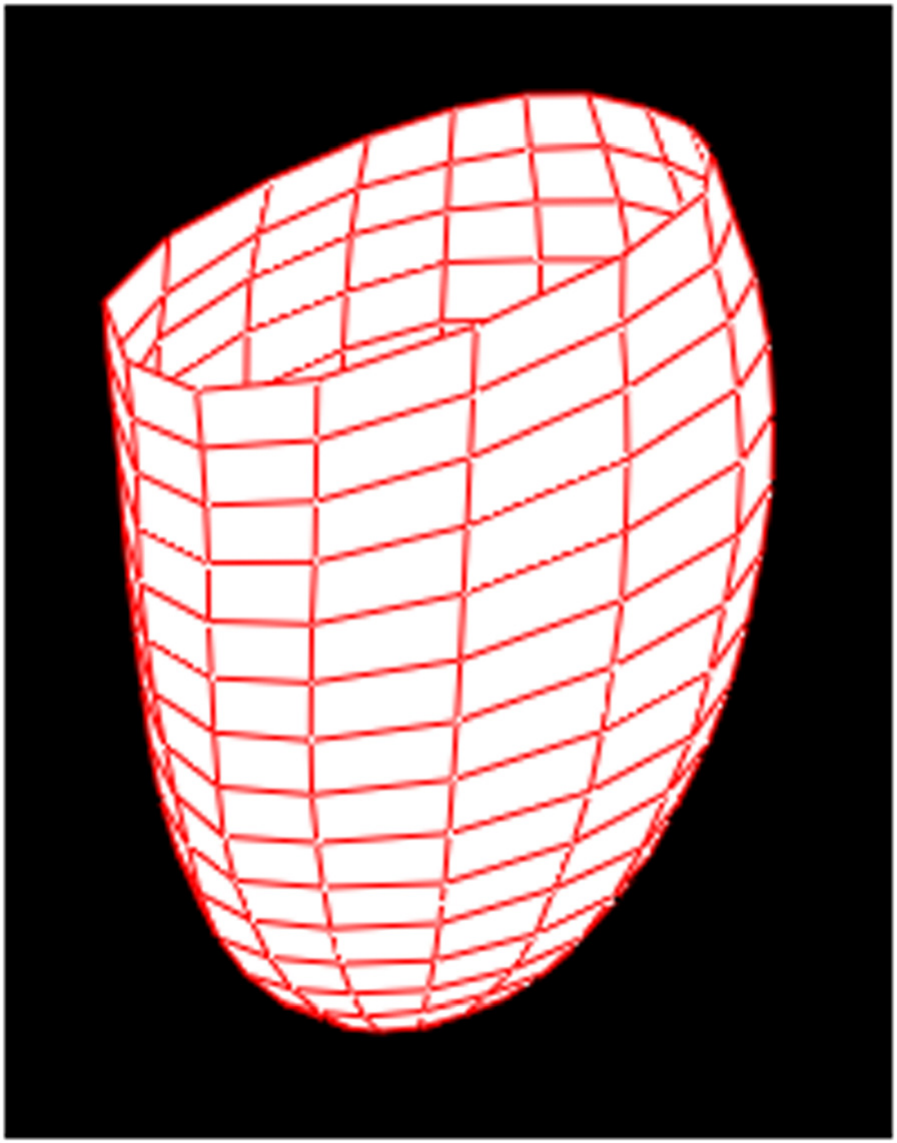
The left ventricle represented by a surface of *rectangles* through the center of the myocardium

The perfusion vector concept is based on a calculation of the difference between the anatomical centroid and the perfusion center of gravity of the left ventricle. The left ventricle is outlined from SPECT images using the EXINI Heart™ software (EXINI Diagnostics AB, Lund, Sweden) and is represented by a surface through the center of the myocardium. This surface is a grid of rectangles where each rectangle is assigned an index *i*, a center position (*x*_*i*,_*y*_*i*_, *z*_*i*_), an area *A*_*i*_, and a weight *W*_*i*_. Figure 1 depicts this grid. Given these definitions, the centroid of the left ventricle myocardium is calculated using Eq. 1. By setting *W*_*i*_ = 1 for all *i*, the formula reduces to the geometrical centroid of the grid. A corresponding perfusion center of gravity is calculated by setting the weights *W*_*i*_ to the measured intensity at each position (*x*_*i*_, *y*_*i*_, *z*_*i*_).

1 $$ C=\left(\frac{{\displaystyle \sum {x}_i{A}_i{W}_i}}{{\displaystyle \sum {A}_i{W}_i}},\frac{{\displaystyle \sum {y}_i{A}_i{W}_i}}{{\displaystyle \sum {A}_i{W}_i}},\frac{{\displaystyle \sum {z}_i{A}_i{W}_i}}{{\displaystyle \sum {A}_i{W}_i}}\right) $$

A perfusion vector is then calculated using Eq. 2.

2 $$ \overrightarrow{\mathbf{P}}={C}_{\mathrm{p}}-{C}_{\mathrm{A}} $$

**Fig. 2:**
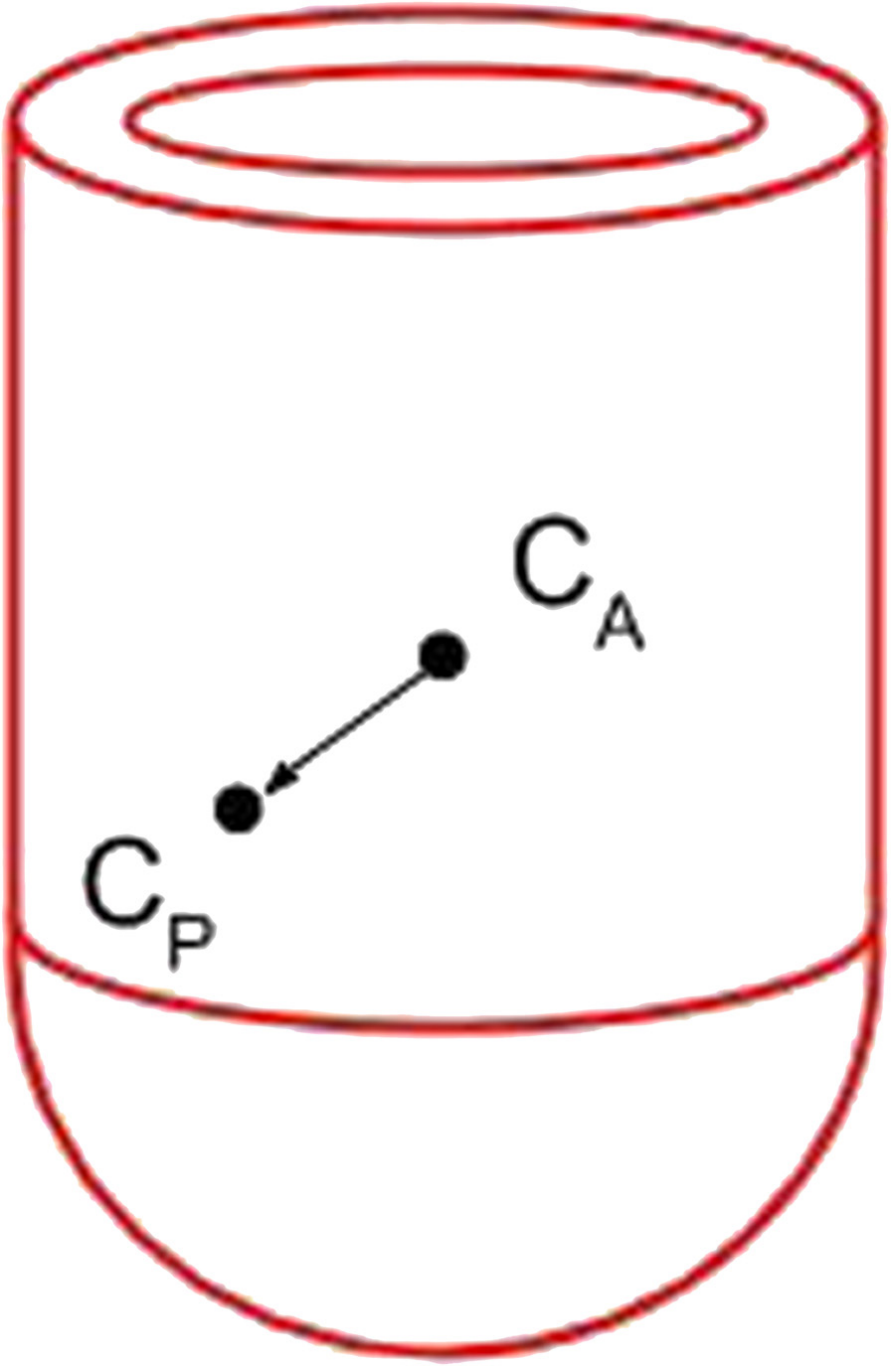
The perfusion vector as the result of the difference between the anatomical centroid, *C*_A_, and the perfusion center of gravity, *C*_P_

**Fig. 3:**
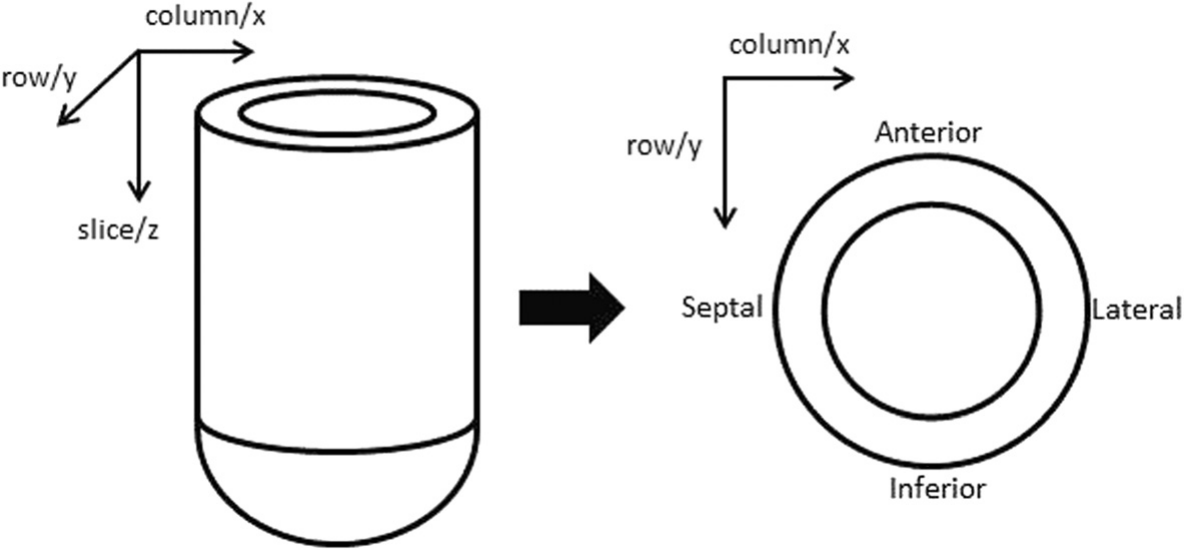
The different axis directions for the perfusion vector

Here, *C*_A_ is the anatomical centroid and *C*_P_ is the perfusion center of gravity. Given a perfusion defect, the vector ^→^**P** would point away from the defect with a magnitude corresponding to the severity and extent of the defect, as shown in Fig. 2. The different axis directions are defined according to Fig. 3. Several different measurements are generated: the size of the perfusion vector at rest and during stress, the direction of the vector (expressed as *x*-, *y*-, and *z*-axes), and the difference between the stress and rest vector magnitudes.

### Simulation study

**Fig. 4:**
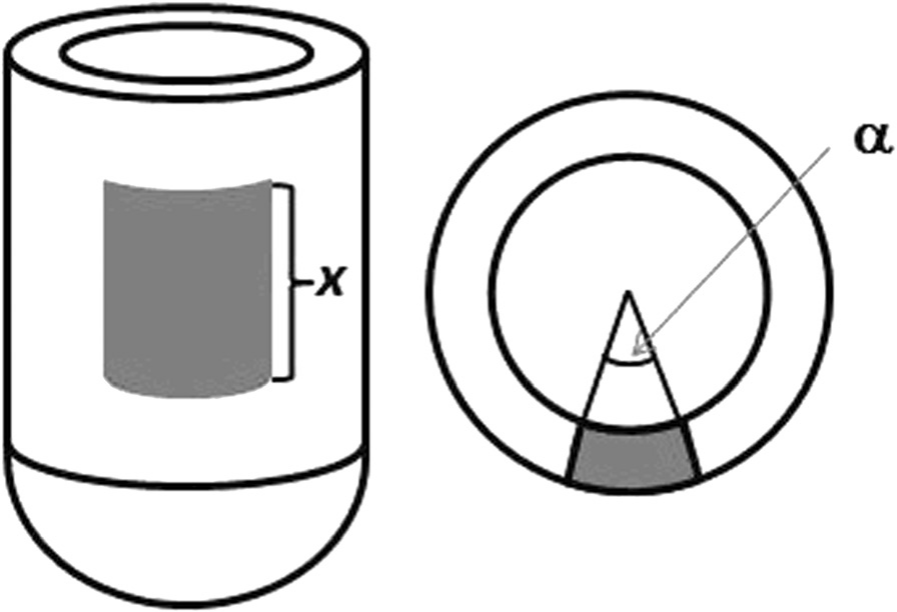
Simulations of perfusion defects. For each defect location (anterior and lateral), four simulations were created with an angle *α* of 10°, 30°, 50°, and 70°, with a propagation in the apical-basal direction (*x*) of 6 cm

The SIMIND Monte Carlo program [11] together with the XCAT phantom [12] was used to simulate projection data. A phantom representing a standard male with an end diastolic volume of the left ventricle of 148 mL was used. To simulate the beating heart and breathing motion, 40 static phantoms were created with the heart in different spatial positions and positioned in the heart cycle. Simulation was performed for each phantom and then merged prior to reconstruction. Perfusion defects were simulated as indicated in Fig. 4. Eight defects were simulated, four anterior angles *α* of 10°, 30°, 50°, and 70°, with a propagation in the apical-basal direction (*x*) of 6 cm, and four lateral defects of corresponding sizes. Simulations without defects were also performed. For each simulation, 32 projections in a 180° arc, starting at 45° right anterior oblique position, were created and a 128 × 128 matrix with a pixel size of 4.8 mm was used.

In order to mimic real measurements, the simulations were performed with enough photons to avoid Monte Carlo noise. Instead, Poisson noise was added after the simulations, corresponding to measurements with an administered activity of 600 MBq, an uptake of 1 % in the heart and where every projection was measured for 20 s. Tomographic images were then reconstructed using the ordered subset expectation maximization (OSEM) algorithm, four iterations, and eight subsets. A Gaussian post filter was applied with a full width at half maximum (FWHM) of 7 mm. Short-axis slices were created from the trans-axial slices. Scatter and attenuation corrections were not applied.

### Study population

One thousand two hundred eighty-three patients referred for MPS in Malmö, Sweden, in 2007 were considered for inclusion. One of the investigators (ET) categorized all patients into the following groups: normal studies, anterior infarction, apical infarction, lateral infarction, inferior infarction, anterior ischemia, apical ischemia, lateral ischemia, inferior ischemia, mixed defects, multiple defects, equivocal studies, and stress-only studies. The categorization was based on visual assessment of the MPS images. Patients with mixed defects, multiple defects, equivocal studies, and stress-only studies were excluded since well-defined defects were desirable in this first patient study. The first 10 patients (40 for normal studies) to be included in the categories were used as the study population. Thus, 80 abnormal and 40 normal studies were included. EXINI Heart™ (EXINI Diagnostics AB, Lund, Sweden) was used to automatically derive the size of ischemia and infarction for all patients based on their artificial neural network algorithm. The study was approved by the local research ethics committee at Lund University and complies with the Declaration of Helsinki.

### MPS protocol

The MPS studies were performed using a 2-day gated stress/non-gated rest Tc-99m-tetrofosmin protocol, starting with injection of 600 MBq Tc-99m-tetrofosmin at stress, followed by 600 MBq Tc-99m-tetrofosmin at rest. Patients were stressed using either maximal exercise on an ergometer or pharmacological test with adenosine.

Stress and rest acquisitions began about 60 min after the injection of Tc-99m-tetrofosmin. Images were obtained according to established clinical protocols, using SPECT over 180° elliptical and autocontour rotations from the 45° right anterior oblique position, with a dual-head gamma camera, e.cam (Siemens AG Medical Solutions, Erlangen, Germany). Patients were imaged in the supine position. Low-energy high-resolution collimator and a zoom factor of 1.0 were used. We obtained 64 projections in a 128 × 128 matrix, with an acquisition time of 25 s per projection. Tomographic reconstruction and calculation of short- and long-axis slice images were performed using e.soft (Siemens AG Medical Solutions, Erlangen, Germany). Images were reconstructed with filtered back projection. A two-dimensional Butterworth pre-reconstruction filter was used with cut-off frequency of 0.45, order 5.

### Statistical analysis

Kruskal-Wallis analyses were performed to investigate differences in perfusion centroids between the normal and abnormal patients. When significance was found, Mann-Whitney analyses were carried out to test for differences. Abnormal patients were compared to the normal patients; thus, multiple tests were performed. We used a Bonferroni correction to give a significance level of 0.0125 when four groups were tested and 0.025 when two tests were carried out. Spearman’s correlation test was performed to investigate differences between defect extent and magnitude vector. All analyses were carried out using MedCalc v 12.7.7 (MedCalc Software bvba, Ostend, Belgium).

## Results

### Simulation study

**Fig. 5:**
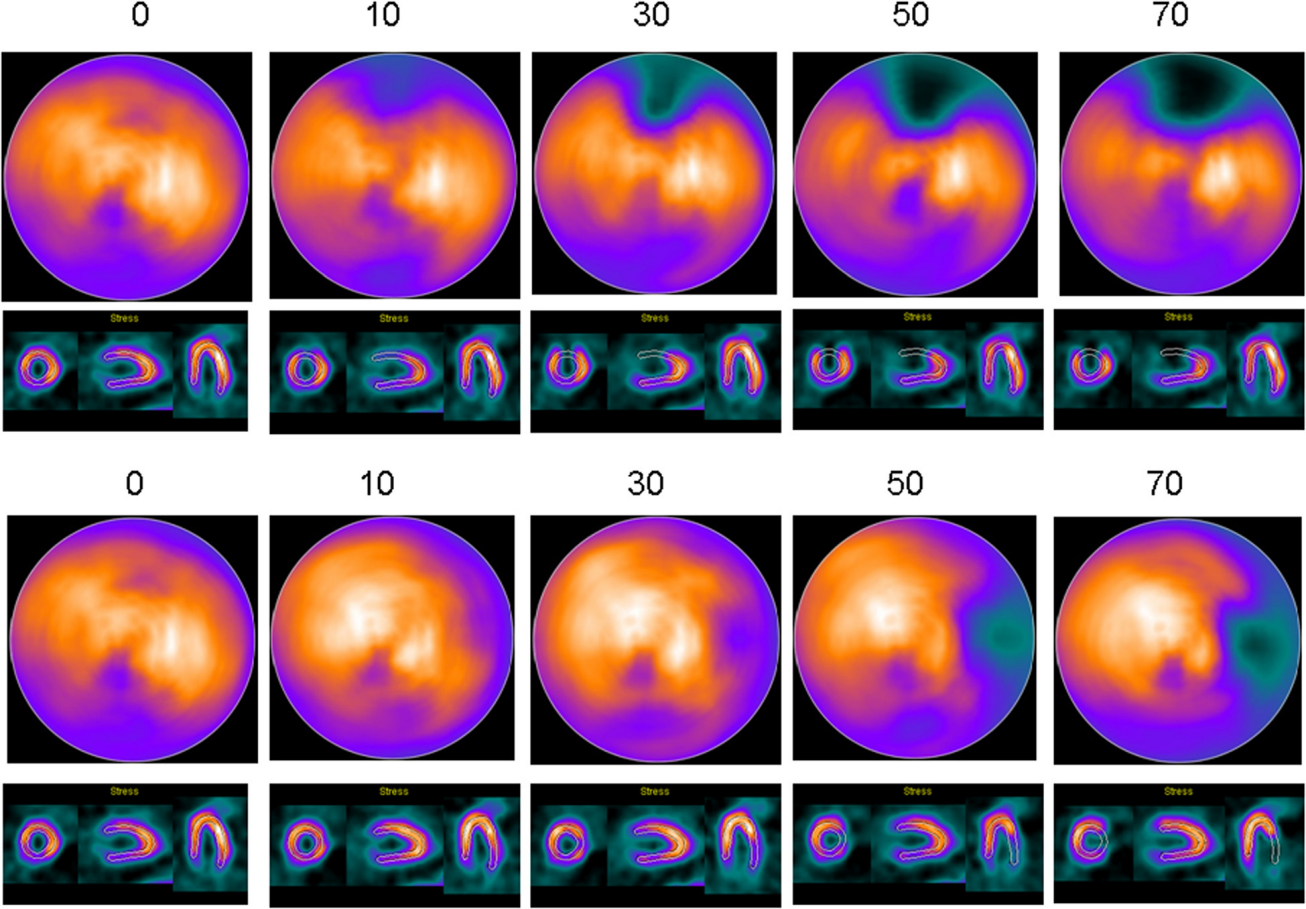
The *upper part* of the figure shows the simulated anterior defects (the first figure without a defect) with an angle *α* of 10°, 30°, 50°, and 70°. The *lower part* of the figure shows the simulated lateral defects

**Fig. 6:**
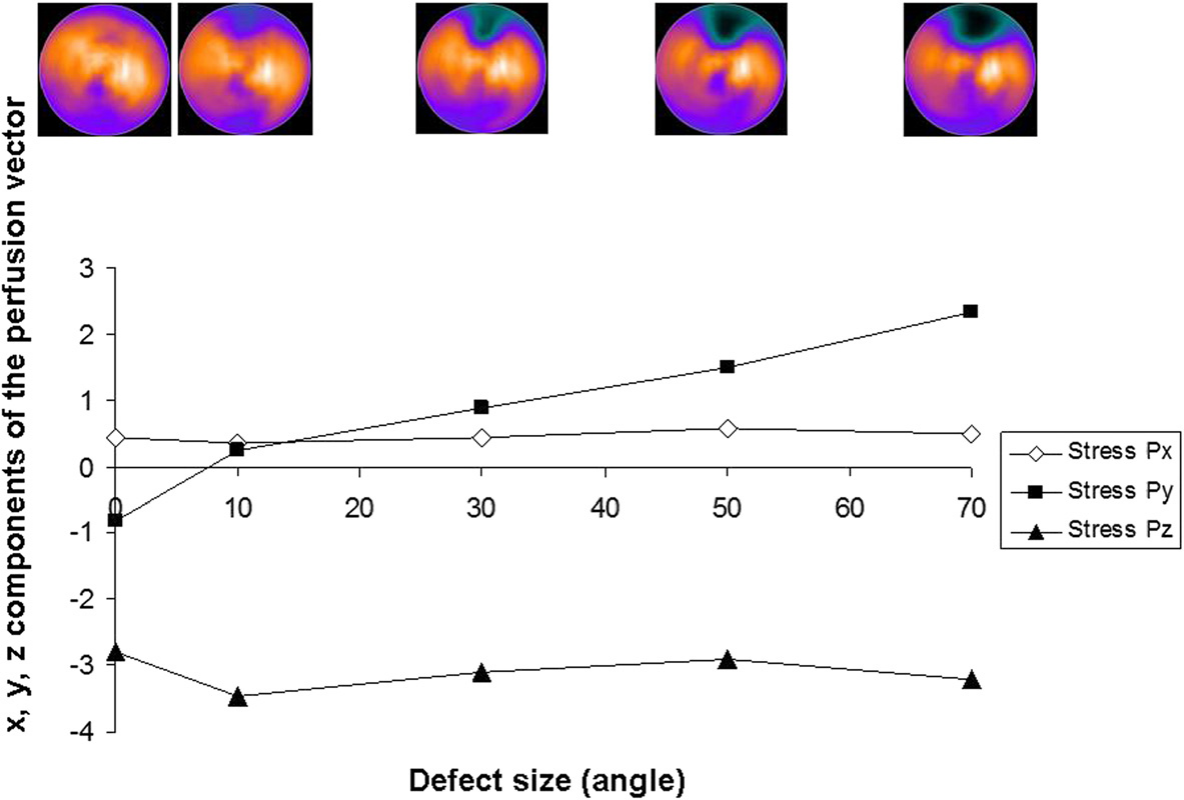
*x*, *y*, and *z* components of the stress perfusion vectors for the different sizes of simulated anterior defects

**Fig. 7:**
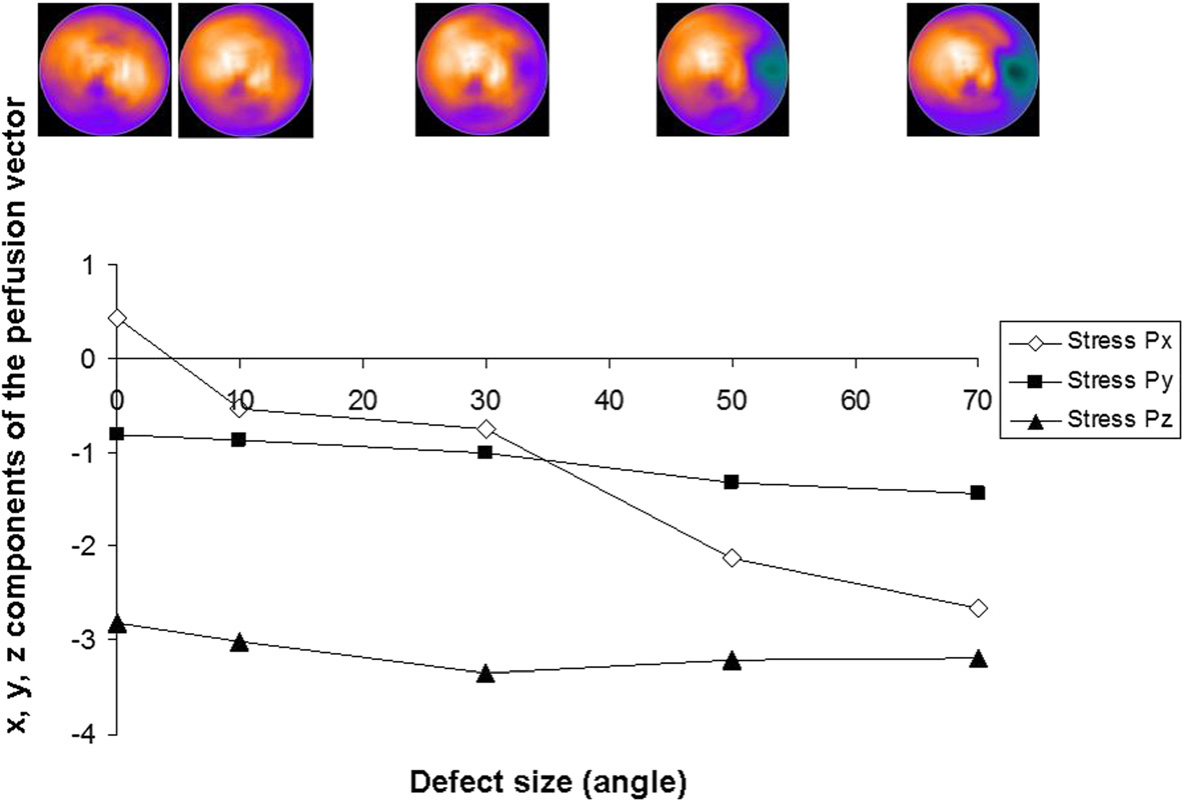
*x*, *y*, and *z* components of the stress perfusion vectors for the different sizes of simulated lateral defects

The simulated MPS images for the different-sized anterior and lateral defects are shown in Fig. 5. For anterior defects, the stress perfusion vector on the *y*-axis (anterior-inferior direction) increased according to the defect size, whereas the vector on the *x*-axis and *z*-axis remained stable (Fig. 6). For the lateral defects, the stress perfusion vector on the *x*-axis (septal-lateral direction) decreased according to the defect size, whereas the vectors on the *y*-axis and *z*-axis remained reasonably stable (Fig. 7).

### Patient study

In the normal patient group, there were 10 men and 30 women. Mean age was 59.3 years (range 35–88 years). For the abnormal patients, 54 men and 26 women were included. Mean age was 65.2 years (range 41–83 years). Mean total extent of defects (automatically derived from EXINI Heart™) was 0.17 % for normal patients (range 0–6.6 %) and 25.0 % (range 0 to 64.4 %) for abnormal patients. For ischemic patients, the mean extent of ischemia was 15.6 % (range 0–37.4 %), and for infarcted patients, the mean extent of infarction was 29.8 % (range 0–64.0 %).

**Fig. 8:**
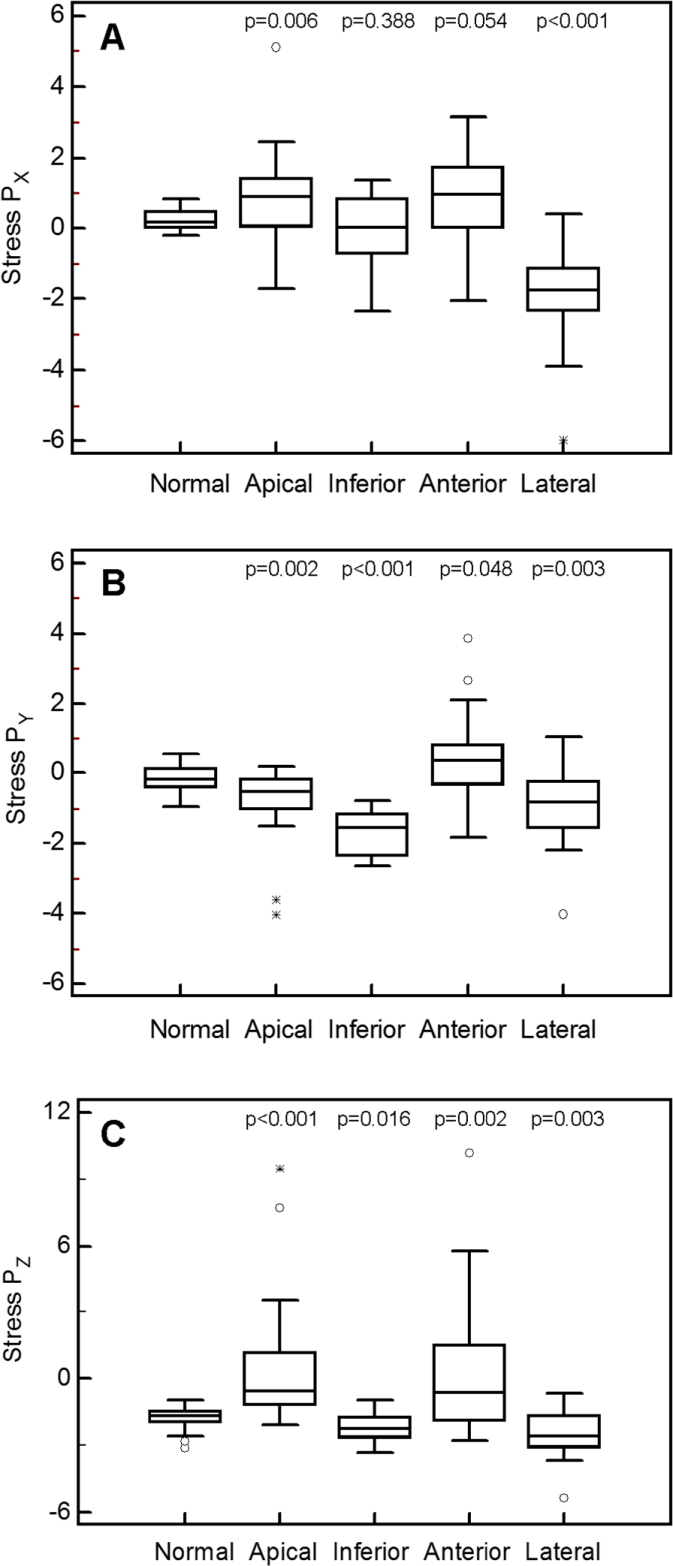
Box plots for the stress perfusion vectors for normal patients and patients with ischemia or infarction at different locations (apical, inferior, anterior, lateral) for the *x*-axis (**a**), *y*-axis (**b**), and *z*-axis (**c**). The *p* value is related to the normal group

**Fig. 9:**
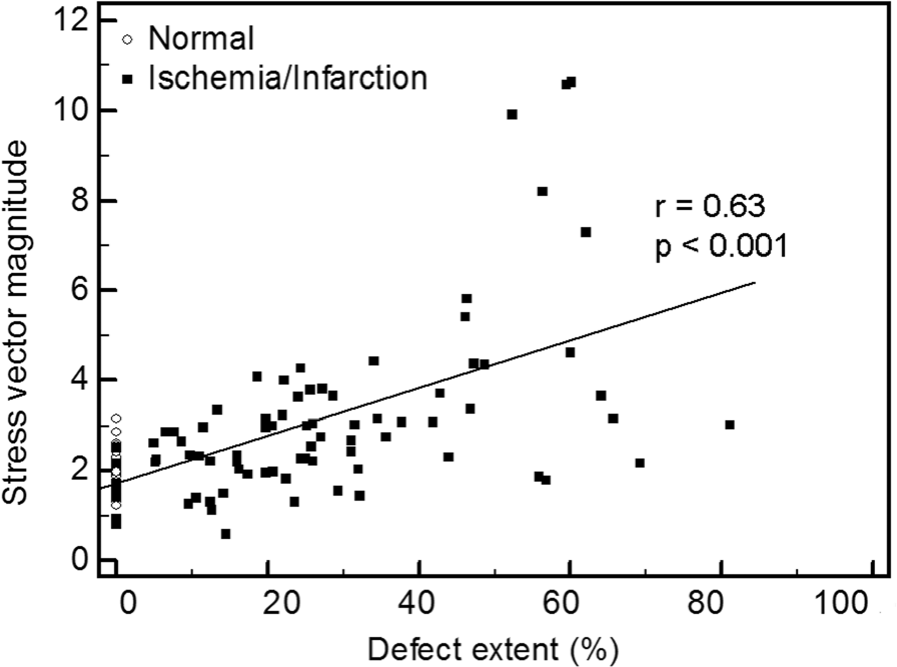
Comparison between stress vector magnitude and automatically derived defect extent

**Fig. 10:**
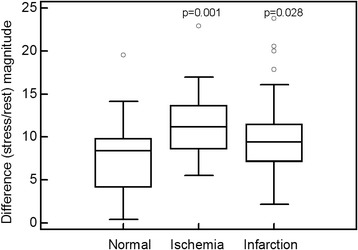
Box plots for the difference magnitude for normal patients, as well as patients with ischemia and infarction. The *p* value is related to the normal group

For the first analysis, stress perfusion vectors were compared between normal patients and four different groups of abnormal patients (apical, inferior, anterior, and lateral ischemia or infarction) for the *x*-, *y*-, and z-axes. As seen in Fig. 8, the range for normal patients was small in all three orientations, whereas the ranges were large in the abnormal patient groups. There was a statistically significant difference between the normal and the apical group and between the normal and the lateral group on the *x*-axis, between the normal and the apical, inferior and lateral groups on the *y*-axis, and between the normal and the apical, anterior and lateral groups on the *z*-axis. The correlation between the defect size and stress vector magnitude (Fig. 9) was found to be statistically significant, with an *r* value of 0.63. There was a statistically significant difference in the difference (rest/stress) vector magnitude between the normal patients and the ischemic patients, but not between the normal patients and the patients with infarction (Fig. 10).

## Discussion

Every day, physicians in radiology and nuclear medicine are faced with interpreting challenging studies. In medical imaging, visual interpretation of images is dependent on the knowledge of the physician and is subject to inter- and intra-observer variabilities. There is a strong need for the development of automatic, quantitative measurements to be used in addition to the visual interpretation in order to help standardize interpretation and facilitate a correct diagnosis.

In this study, we wanted to develop an automatic, quantitative measurement in MPS studies, which could assist the visual interpretation. We therefore introduced the concept of perfusion vector, based on the difference between the anatomical and perfusion-weighted centroid. Based on the simulation studies, we found that for anterior defects, the perfusion vector changed in the anterior-inferior direction, in proportion to the size of the defect. For the lateral defects, the perfusion vector changed in the septal-lateral direction, in proportion to the defect size.

After these encouraging simulation results, we wanted to validate the method in a patient population. The stress perfusion vector changed drastically for the patients with lateral defects on the *x*-axis, as expected. The vector for the apical defects (which often is oriented towards the septal-anterior part of the left ventricle) changed in the opposite direction. For the *y*-axis, the change of the perfusion vector for the inferior defects was oriented in the opposite direction compared to anterior defects (not statistically significant compared to normal patients). We found a significant difference also for patients with apical defects and lateral defects. Lateral defects are often located slightly in the inferior part of the left ventricle and apical defects often slightly in the anterior part of the left ventricle, which could explain these results. Finally, for the *z*-axis, as expected, the perfusion vector for the apical defects was significantly different from the normal patients. The change in perfusion vector on the *z*-axis for the anterior, inferior, and lateral defects will depend on if the defects are located in the basal or apical part of the left ventricle. Patients with apical defects were included as a separate group (although often located towards the anterior part of the left ventricle) in order to see if changes in the *z*-axis were present.

The difference (stress/rest) vector could be used to differentiate between patients with and without ischemia, but there was a large inter-individual variability. The vector magnitude correlated quite well with the automatically derived extent of the defects.

There are several possible reasons for the large inter-individual variability in the abnormal patients. The patients were selected only based on the main localization of the defect, not based on the size of the perfusion defects. Therefore, both small, medium-sized and large defects are included in the material, and the mean defect size is likely to be different for different groups. Also, patients tend not to have “perfect” distribution of tracer in areas without perfusion defects. For example, if the delineation of one part of the ventricle is slightly outside the ventricle border, there is a lower uptake in one (or more) of the basal part(s) of the ventricle, which affects the calculated perfusion vector.

The most encouraging result from the patient study was the small variation in all three orientations for the normal patients compared to the abnormal patients. It is possible that the method could be useful for the determination if a study is normal. This could for example facilitate the determination if a rest study is needed after a stress study, but this needs to be further tested. In this study, no attenuation-corrected images were studied. It is unknown how the perfusion vector method works in attenuation-corrected images, but one hypothesis is that the variation in different orientations for the normal patients will be even smaller due to a more homogenous tracer uptake in attenuation-corrected images. An advantage of the perfusion vector concept is that the method is fully automated and thus eliminates subjective assessment of the images.

### Study limitations

For the normal group, we used patients that were admitted for clinical MPS due to suspicion of coronary artery disease but were interpreted as normal. It would be preferred to use a normal population, with a low likelihood of coronary artery disease. Only a limited number of patients with ischemia and infarction were included. We also do not have any other method, such as invasive coronary angiography with fractional flow reserve measurements, to validate the interpretation of SPECT images.

## Conclusions

The concept of the perfusion vector introduced in this study is shown to have potential in assisting the visual interpretation in MPS studies. The small variation in normal patients compared to the abnormal patients could prove to be helpful to determine if a study is to be considered normal and without perfusion defects. For the abnormal studies, there was a gradual change of the perfusion vector correlating to the size of the perfusion defect, both in simulated images and in patient studies. Hence, here, the perfusion vector could assist when assessing the size of a perfusion defect. Further studies are needed to validate the concept in patients.
